# Genome maintenance and bioenergetics of the long-lived hypoxia-tolerant and cancer-resistant blind mole rat, *Spalax*: a cross-species analysis of brain transcriptome

**DOI:** 10.1038/srep38624

**Published:** 2016-12-09

**Authors:** Assaf Malik, Vered Domankevich, Han Lijuan, Fang Xiaodong, Abraham Korol, Aaron Avivi, Imad Shams

**Affiliations:** 1 Institue of Evolution, University of Haifa, Haifa 3498838, Israel; 2 Bioinformatics Core Unit, University of Haifa, Haifa 3498838, Israel; 3 Deparment of Evolutionary and Environmental Biology, University of Haifa, Haifa 3498838, Israel; 4 BGI-Tech, BGI-Shenzhen, Shenzhen 518083, China

## Abstract

The subterranean blind mole rat, *Spalax,* experiences acute hypoxia-reoxygenation cycles in its natural subterranean habitat. At the cellular level, these conditions are known to promote genomic instability, which underlies both cancer and aging. However, *Spalax* is a long-lived animal and is resistant to both spontaneous and induced cancers. To study this apparent paradox we utilized a computational procedure that allows detecting differences in transcript abundance between *Spalax* and the closely related above-ground *Rattus norvegicus* in individuals of different ages. Functional enrichment analysis showed that *Spalax* whole brain tissues maintain significantly higher normoxic mRNA levels of genes associated with DNA damage repair and DNA metabolism, yet keep significantly lower mRNA levels of genes involved in bioenergetics. Many of the genes that showed higher transcript abundance in *Spalax* are involved in DNA repair and metabolic pathways that, in other species, were shown to be downregulated under hypoxia, yet are required for overcoming replication- and oxidative-stress during the subsequent reoxygenation. We suggest that these differentially expressed genes may prevent the accumulation of DNA damage in mitotic and post-mitotic cells and defective resumption of replication in mitotic cells, thus maintaining genome integrity as an adaptation to acute hypoxia-reoxygenation cycles.

The blind mole rat of the genus *Nannospalax* (hereafter, *Spalax*) is a subterranean, hypoxia tolerant rodent, evolutionarily related to murines. The last common ancestor of *Spalax*, mouse, and rat lived ~46 million years ago^1^. Despite the tight evolutionary relatedness of *Spalax* and murines, they exhibit profound differences in lifespan, propensity to cancer diseases^2^, behavior, sensory-perception, and susceptibility to different stresses^3^,^4^,^5^. Although a very common cause of death in rats and mice is cancer^6^, *Spalax* resists experimentally induced carcinogenesis *in vivo* and does not develop spontaneous cancer^2^. While both rat and *Spalax* have comparable body weights, their maximum lifespan is ~4 years and ~20 years, respectively^7^. The naked mole rat (*Heterocephalus glaber)*, another hypoxia-tolerant subterranean species of the *Bathyergidae* family, separated by ~85 million years of evolution from *Spalax*^1^, is also long-lived, and was reported to be less sensitive to spontaneous cancers^8^, except few cases recently reported^9^. Molecular adaptations to subterranean life and longevity where suggested for this species, in a brain transcriptome study^10^. Noteworthy, we have proved that both *Spalax* and naked mole rat’s normal fibroblast secrete substance/s interacting with cancer cells from different species, including a wide variety of human cancer cells, ultimately leading to the death of the cancer cells^2^. In addition, sequence similarities between distantly related hypoxia-tolerant species (diving- and subterranean- mammals) were found in the protein sequence of p53^11^, a master regulator of the DNA damage response (DDR). These studies indicate that adaptations to hypoxia include changes in the DDR that may be linked to cancer resistance, and longevity traits.

Under laboratory conditions, *Spalax* survives ~3% O_2_ for up to 14 hours, whereas rat survives such conditions for only ~2–3 hours. Oxygen levels measured in *Spalax*’s natural underground burrows vary between ~21% and 7%, depending on seasonal and ecological conditions^3^,^5^,^12^. In its natural habitat, *Spalax* is exposed to acute and transient hypoxia, such as: (i) long-term periods of hypoxia during seasonal rainfalls, which reduce soil permeability to oxygen, and simultaneously reduce the total space available to the animal^12^,^13^; and (ii) short-term periods of hypoxia during extensive digging activity, when burrows are clogged by soil pushed to the rear by the animal, forcing it to perform an energy-consuming activity in a small burrow fragment with a limited amount of oxygen^12^. Hence, in its natural habitat*, Spalax* faces acute cyclical changes in oxygen levels. By the term “acute hypoxia” we refer to short- or long- term hypoxia for a limited period, followed by reoxygenation, which is in contrast to “mild-chronic hypoxia” characterizing habitats, such as high altitudes.

At the cellular level, cycles of acute hypoxia followed by reoxygenation may act as a driving force of genomic instability, which underlies both aging and cancer. Severe hypoxia induces S-phase arrest due to the inhibition of DNA synthesis in a HIF1-independent manner^14^. Hypoxia leads to dNTPs depletion^14^, and since dNTPs are required for replication, this condition increases replication-stress^15^, a condition defined as the stalling or slowing of replication fork progression and/or DNA synthesis^16^,^17^. In the case of short-term hypoxia (less than 12 hours), cells are still capable of restarting replication within two hours following reoxygenation^14^. Under reoxygenation, replication restarts in the presence of reactive oxygen species (ROS)-induced oxidative damage, leading to the accumulation of DNA lesions. Moreover, this happens when the DNA repair pathways, which are required for overcoming oxidative damage and replication stress [e.g., homologous recombination (HR), mismatch repair (MMR), and base excision repair (BER)], are not yet recovered from the repressive effect of hypoxia^18^. Accordingly, it was found that repression of DNA repair under hypoxia could lead to genome instability deterioration during re-oxygenation^18^,^19^. A similar process was described in cancer cells, in which the increase in genomic instability was attributed to fluctuations in oxygen levels evidenced in the tumor microenvironment during cyclic events of angiogenesis^19^,^20^.

The *Spalax* genome was recently sequenced^21^, and *Spalax* normoxia vs. hypoxia genome-wide molecular responses were explored in both brain and muscle tissues^21^,^22^. These studies revealed the involvement of multiple functional groups of genes in the hypoxia-induced responses of *Spalax*. For example, it was shown that hypoxia induces genome-wide change in transcript abundance of a remarkably large number of genes associated with apoptotic regulation, including anti-apoptotic response and cancer-related genes, in *Spalax* brain and muscle tissues^22^. In cells with intact p53, hypoxia-related conditions may lead to the activation of apoptosis^14^. *Spalax*’s wild type p53 demonstrates impaired apoptotic function and increased cell cycle arrest/DNA repair compared to the human p53^23^. This adaptation may serve as a strategy to avoid multiple events of cellular apoptosis, providing the cell an ‘opportunity’ to repair, recover, and avoid progenitor cell pool depletion that may eventually lead to early aging^24^. However, impaired p53 apoptotic functions under oxygen fluctuations may also increase the risk of DNA-defected cell escape and consequently lead to cellular transformation^14^. The observations that *Spalax* does not develop spontaneous cancer and exhibits an extraordinary long life-span led us to hypothesize that it has evolved unique mechanisms to cope with the above-mentioned threats and to guard its genome under the harsh conditions of acute oxygen fluctuations.

The major goal of this study is to compare whole brain transcript abundance profile of *Spalax* to this of its phylogenetically related *Rattus* (hereafter, rat). The mammalian brain is a highly oxygen-dependent organ that consumes high percentage of the body oxygen relative to its size (in humans, about 20% of the oxygen consumption and 2% of body weight)^25^. The brain is highly sensitive to hypoxia, and in humans it is related to several ageing disorders, such as Alzheimer, Parkinson, and stroke^26^,^27^,^28^. The brain includes both dividing cells, such as astrocytes and glial cells, and post mitotic cells such as neurons that accumulate DNA damage along lifespan. Lack of oxygen in the mammalian brain usually causes fast, severe and irreversible damage^29^. However, this is clearly not the case in the brains of different diving-30,31 and subterranean-mammals^32^ that may survive recurrent and extended periods of systemic hypoxia without damage. We accordingly hypothesize that evolutionary adaptations associated with hypoxia tolerance include changes in the regulation of DNA repair pathways in the *Spalax* brain transcriptome. By employing RNA-Seq approach, we compared *Spalax* and rat orthologous gene expression in brain tissues. We developed a computational procedure which reduces RNA-Seq technical biases in cross-species analyses, a problem that, to our knowledge, was handled only by few RNA-Seq studies before^33^. We found that, compared to rat, *Spalax* maintains significantly higher mRNA levels of genes associated with DNA damage repair and functionally related DNA metabolic pathways. At the same time, we found lower mRNA levels of genes whose products act in the mitochondria and are related to oxidative phosphorylation, a metabolic process that produces ROS, which at excessive levels cause cellular damage and contribute to disease and aging. Based on the foregoing functional profiles, we suggest that the observed interspecies changes in transcript abundance are associated with genome stability in *Spalax*; and therefore, contribute to cancer- and hypoxia-tolerance and longevity.

## Methods

### Animals

The blind mole rat, *Spalax*, is a wild, solitary animal, and the Institute of Evolution, University of Haifa, Israel is certified by the Israel Nature and Park Authority, Science and Conservation Unit, to capture and euthanize *Spalax* individuals for scientific studies. *Spalax* individuals were captured in the field, and housed in the Animal House of the Institute of Evolution, University of Haifa, Israel, under permission of the Ethic Committee - Institutional Review Board to Evaluate Animal Subject Research of the University of Haifa (Reference # 318/14). Considering *Spalax* innate solitary nature, they are housed in individual cages (45 × 30 × 20 cm) filled with a bed of saw-dust, allowing their natural behavior of fossorial activity. *Spalax* individuals are fed mainly with carrots and potatoes. Food and saw-dust are refreshed twice a week. *Spalax* are housed for three acclimatization months before used in experiments. The Animal House is kept under controlled conditions of 22–24 °C with seasonal light/dark hours, and all animals are subjected to routine veterinary inspection by the Institutional Veterinarian. All rats individuals (Sprague Dawley) used in this study were purchased from the Israeli branch of Harlan Laboratories Inc. Animals used in this study were adults of different ages. All experiments were performed in accordance with relevant guidelines and regulations of the University of Haifa. All experimental procedures including maintenance, euthanizing by an inhaled overdose of isoflurane and sacrificing, harvesting tissues, tissues’ storage and experimental manipulations have all been approved by the Ethic Committee - Institutional Review Board to Evaluate Animal Subject Research of the University of Haifa (Reference # 193/10).

### Experimental settings

Our main goal was to identify genes whose mRNA abundance differ between the brains of *Spalax* and their evolutionarily related aboveground rats, using RNA-Seq. Due to the large lifespan differences between *Spalax* (~20 years) and rat (~4 years), it is difficult to determine comparable time points (ages). Thus, we used samples from a wide range of ages (see experimental design, Supplementary Table S1), and searched for genes whose transcript abundance is consistently upregulated or down regulated compared to rat. Since *Spalax* cannot be bred in captivity, and animals that are captured in the field are kept in an animal house, specimen choice is dictated by the availability of individuals of different sexes. As shown in Supplementary Table S1, the obtained data include samples belonging to different sexes, or samples sequenced in different sequencer settings. Since we are interested in estimating the effect of the species factor on the transcript abundance, we verified that the contribution of additional factors is limited by validating observed RNA-Seq interspecies differential expression calls using quantitative real-time PCR (see results).

### RNA extraction and sequencing

RNA was extracted from tissues, flash-frozen in liquid nitrogen, and stored in −80 °C. *Spalax* and rat brain RNA was extracted from the whole brain by using TRI Reagent (Molecular Research Center, Cincinnati, USA) following the manufacturer’s instructions. All samples were quantified on a Nanodrop^®^ spectrophotometer and quality assessed on the Agilent Bioanalyzer. The strand-specific RNAseq libraries were prepared with Illumina’s TruSeq Stranded RNA Sample Prep kit according to the manufacturer’s protocols. For qPCR, RNA samples were treated with DNase I (DNA-free, Ambion), and 1 μg was taken for first-strand cDNA synthesis (iScript, Bio-Rad) in a 20-μl volume. Aliquots of 1 μl of cDNA were used for each real-time PCR reaction.

### RNA-Seq quality filtering and mapping

After obtaining the sequencing data, Paired End reads (PE) were cleaned from adapters and low-quality regions, using Trimmomatic 0.3^34^. The quality of reads was further inspected using Fastqc (www.bioinformatics.babraham.ac.uk). Quality filtered fastq file pairs were deposited in the NCBI Sequence Read Archive (SRA), accessions: SRR5039063, SRR5039065, SRR5039059, SRR5039064, SRR5039061, SRR5039066, SRR5039062, SRR5039058, SRR5039057, SRR5039055, SRR5039056, SRR5039060. *Spalax* and rat RNA-Seq reads were first mapped to their respective genomes, using STAR 2.4^35^, in two passes: in the first pass reads were aligned to the genome using STAR default parameters, while in the second pass splice-junctions retrieved from the first pass were used. Reads’ alignments were visualized using IGV, and correct mapping to exons was verified^36^.

### RNA-seq validation and gene quantification

To validate RNA-seq results, absolute gene quantification was performed using StepOne (Applied Biosystems) Quantitative Real-Time PCR. The cDNA of 3 adult rat and 3 *Spalax* individuals (Supplementary Table S1) were used to quantify transcripts levels of seven representative genes: *POLA2, BRCA2, UNG, TOP3A, TDP2, NIT1, SLC25A23*; where the first five genes were identified as up-regulated in *Spalax* relative to rat according to RNA-seq analysis, and the two latter genes as down-regulated. Primers were designed, using Primer Express 3/2 software (Applied Biosystems). As we did not succeed to design identical primers for both species, two separate standard curves were employed based on known quantities of a single -stranded oligonucleotide corresponding to the expected PCR product. Gene expression was normalized to ribosomal RNA and to total RNA, which is recommended as the most reliable method in the absence of a proven housekeeping gene^37^. Primers for 18S ribosomal RNA were identical for both species (Supplementary Table S2). Gene expression rates are given in copies of cDNA generated from 50 ng of total RNA. Reactions were performed in triplicates using FAST SYBR^®^ Green PCR Master Mix (Applied Biosystems). Dilutions of cDNA were used in order to verify efficiency of the PCR. For ribosomal RNA, cDNA was diluted 1:1000. The amplification parameters were according to the machine default setup as follows: 95 °C for 20 sec, followed by 40 cycles of 95 °C for 3 sec and 60 °C for 30 sec. To verify single product with fixed melting temperature, melting curve protocol was applied. Welch’s t-test was used to statistically test the significance of the differences between *Spalax* and rat transcript abundance.

### Cross-species annotation using RNA-Seq data

*Spalax* vs. rat putative orthologous genes (1:1 orthologs only) were detected by Inparanoid^38^, using rat Ensembl protein sequences (www.ensembl.org) and *Spalax* protein sequences^21^. We then prepared cross-species genomic annotation data for *Spalax* vs. rat. This was done as follows: **(1)** pairwise alignments of *i* orthologous transcripts (*i* = 13,000) were built using MAFFT (http://mafft.cbrc.jp); **(2)** every alignment, *a*_*i*_, was divided into *j* 25 bp sub-alignments; **(3)** for each sub-alignment, *a*_*ij*_, with >70% identity, and <3 bp gaps, the matching genomic regions *g*_*ij,species1*_and *g*_*ij,species2*_ were retrieved and stored in gtf format; **(4)** for each gene, RNA-Seq reads coverage levels in all *g*_*i,species1*_and *g*_*i,species2*_ genomic sub-regions were calculated using the Bedtools option coverageBed (For example, Supplementary Fig. S1 demonstrates the interspecies coverage differences for the genes *BRCA2* and *NIT1*); **(5)** we excluded orthologous genes whose coverage along the gene was poorly correlated between the species (*r* < 0.4), unless the coverage was consistently higher in one species compared to the other (Sign test *p*-value < 0.001, using R binom.test function); **(6)** for each gene *i*, we plotted the coverage relationship between all j sub-alignments in *g*_*i,species1*_vs. *g*_*i,species2*_ and then calculated linear regression line (RLM module in R). RLM outliers were excluded, unless the sign test was significant (see previous step); **(7)** two final coordinates-files in gtf format were produced, one for each species, after excluding the above-mentioned incomparable genes and gene regions.

After implementing the above pipeline, all sub-alignments of all output genes had >70% identity, with <3 bp gaps. Coverage plots were visually inspected using IGV^36^ and visualization scripts.

### Cross-species differential expression analysis

Read counts per gene were calculated using Htseq-count^39^, based on the genome annotation gtf-files produced for comparable regions across-species (as above) and STAR reads alignments. We conducted a cross-species differential expression analysis (DE) of *Spalax* vs. rat (brain samples), using EdgeR^40^ and Deseq2^41^. Both programs gave similar results. In EdgeR, prior to DE analysis, TMM (The trimmed mean of M-values normalization method) normalization was conducted^42^, and CPM (count per million) and FPKM (Fragments Per Kilobase of transcript per Million mapped reads) values were obtained. Both EdgeR TMM and Deseq2 default normalizations assume that the counts of up- and down-regulated genes are comparable, though both are robust for a considerable level of asymmetry. In the tested interspecies data, EdgeR and Deseq2 interspecies MA plots appeared symmetric. Since only comparable gene regions were considered, with nearly equal lengths, normalization by gene length was not needed for cross-species DE analysis in EdgeR and Deseq2. In order to perform functional enrichment analyses, we first selected the group of all significantly DE genes (adj. *p* value <0.05) with 1.0 > log_2_FC as a foreground (or <−1.0), and all tested genes as a background (those genes used by EdgeR for DE genes analysis). Enrichment analysis was conducted using the tools Cluster-Profiler^43^ in R, GoSeq in R^44^, as well as the online tools g:profiler^45^, and Gorilla^46^. GoSeq can be used with Wallenius selection bias correction, or without such correction (assuming no length/read-count bias). It is recommended to use the Wallenius method with all DE genes as the foreground, rather than genes at specific cutoff, and therefore we report here the GoSeq results without bias correction. At the same time, Wallenius correction has not changed the major significance trends. We have searched for functional enrichment using a foreground group of all significantly DE genes (with no fold change cutoff) using the tool LR-Path RNA-enrich^47^, which corrects for read-count selection bias, and its setting allows considering up/down regulated genes separately. According to this tool, low or no read-count selection bias is seen in the data we tested. All above R analyses were conducted in R 3.1.2 (https://www.r-project.org). Pathview^48^ R package was used to illustrate DE genes fold change values on KEGG maps.

## Results

### *Spalax* vs. rat comparable gene annotation for RNA-Seq analysis

Changes in RNA-Seq coverage between different regions of the same gene, or between homologous regions across species, frequently stem from technical RNA-Seq biases^33^,^49^,^50^,^51^. Since orthologous genes frequently differ in transcription start/end sites and the number of exons, we ignored all regions of orthologous genes with low or no sequence similarity between species (see Methods). After implementing this filtering procedure, in all orthologous genes the total size of compared regions was nearly equal in both species. In our data we saw that RNA-Seq reads coverage levels, when measured at consequent intervals along the tested gene sequence, are highly correlated between samples belonging to the same species and tissue. We also observed that these coverage relationships are best explained using linear regression. We further tested the frequency distribution of the average coverage correlation between RNA-Seq samples belonging to the same tissue (i.e., brain) and condition (i.e., normoxia), but to two different species, rat vs. *Spalax* (Supplementary Fig. S1). As shown in Supplementary Fig. S2, the interspecies Pearson’s correlation histogram peaks at around *r* = 0.75. Accordingly, the level of interspecies coverage correlation was used as one of several criteria, in order to decide which genes can be analyzed (see Methods). In total, out of ~13,000 orthologous gene pairs considered, 10,031 pairs were found to be comparable for the DE analysis.

### Intensive *Spalax* vs. rat up/down-regulation of transcripts associated with genome maintenance and bioenergetics, respectively

Differentially expressed (DE) genes were identified in *Spalax* vs. rat whole brain using EdgeR, and those with an adjusted *p*-value <0.05 were defined as significant. Of 10,031 genes whose transcript abundance was detectable in the brain of *Spalax* and rat individuals, 1590 were upregulated in *Spalax* compared to rat (at least two fold higher, i.e. log_2_FC > 1), and 1408 were downregulated (log_2_FC < −1). We then compared the frequency of different functional profiles in all 10,031 tested genes, and in the genes that were up-regulated or down-regulated. This was done by performing a functional enrichment analysis using four different tools: Goseq^44^, G-profiler, Cluster-profiler, and Gorilla (Table 1, Supplementary Table S3, Supplementary Table S4). In general, these different tools tend to report the same major trends in most cases. Similarly, we searched for enrichment patterns (see Methods) among all significantly up-/down-regulated genes (no defined FC cutoff), using the tool LR-Path RNA-enrich (Table 2, Supplementary Table S3).

**Figure 1:**
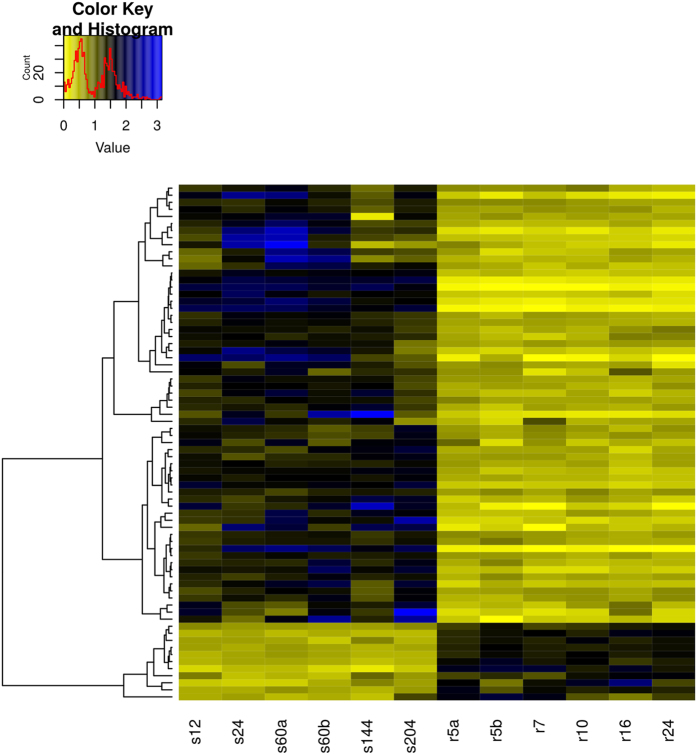
Heatmap representation of transcript abundance differences among genes belonging to the DNA repair functional group, between *Spalax* and rat individuals. Each heatmap’s cell shows the division of an individual FPKM from the mean FPKM of all individuals. Rows represent repair genes, significantly up- or down-regulated (fold change >2 or < −2, adj. P value < 0.05), in *Spalax* vs. rat. The columns represent individuals, labeled “r”, and “s”, (rat and *Spalax*, respectively), sorted by their species and ages. DNA repair genes were significantly overrepresented among *Spalax* upregulated genes (Table 1).

**Figure 2:**
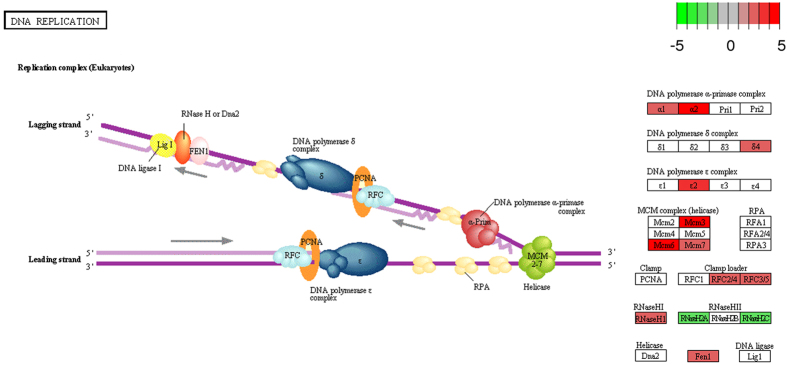
Significant overrepresentation of genes involved in DNA replication, among *Spalax* upregulated genes, compared to rat. Among upregulated *Spalax* genes (fold change >2, adj. P value < 0.05), compared to rat, a significant overrepresentation of genes involved in DNA replication is seen. Larger than two fold upregulated/downregulated *Spalax* genes are superimposed on the significantly enriched DNA-replication pathway of rat (rno03030, obtained by KEGG, Kanehisa Laboratories), and are shown in green to red scale (mapped to negative to positive fold changes values, namely down and up regulation, respectively).

**Figure 3:**
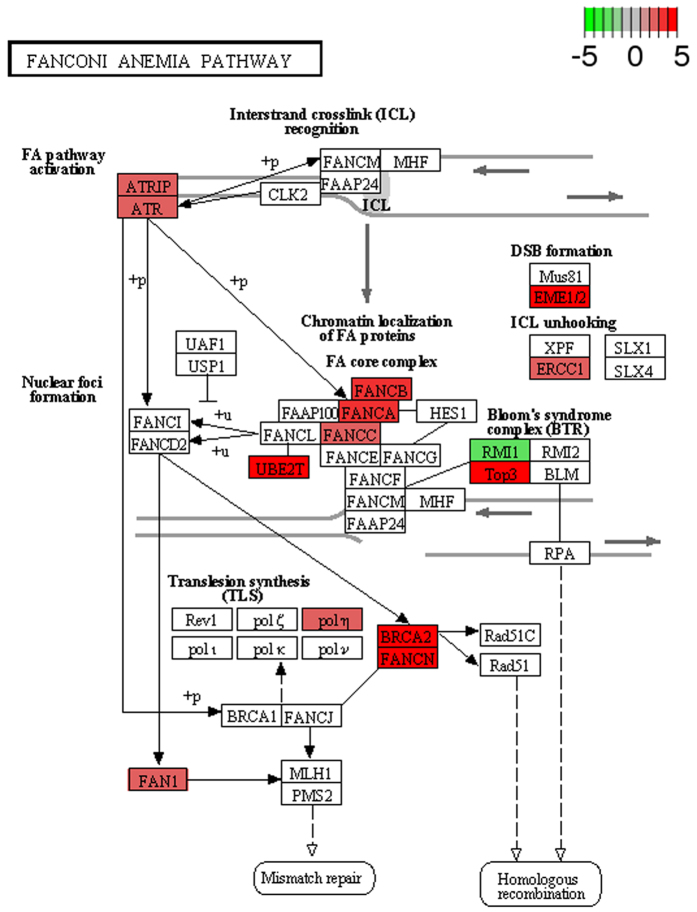
Significant overrepresentation of genes involved in the Fanconi Anemia pathway, among *Spalax* upregulated genes, compared to rat. Among upregulated *Spalax* genes (fold change >2, adj. P value < 0.05), compared to rat, a significant overrepresentation of genes involved in the Fanconi Anemia pathway is seen. Larger than two fold upregulated/downregulated *Spalax* genes are superimposed on the significantly enriched DNA-replication pathway of rat (rno03460, obtained by KEGG, Kanehisa Laboratories), and are shown in green to red scale (mapped to negative to positive fold changes values, namely down and up regulation, respectively).

Among the up-regulated genes, at >2 fold change (Supplementary Table S5), in the *Spalax* brain compared to the rat brain, several functional groups of genes are significantly overrepresented. We assembled significantly enriched functional groups into three supergroups representing related biological functions (Table 1). The logic behind this functional clustering is that: 1) it is known from the biological literature that these functional groups have complementary functional roles; 2) the proportion of genes shared between groups is larger than expected. The first supergroup can be defined as the DNA maintenance supergroup, and it includes functional groups associated with the response to DNA damage (79 genes), DNA-repair (59 genes), DNA replication, DNA metabolism (102 genes), and the Fanconi Anemia pathway (13 genes). In most DE genes belonging to the tested functional terms, interspecies differences are consistent between all individuals, regardless of their ages. For example, as the heatmap in Fig. 1 shows, in genes involved in DNA repair, although a limited effect of age seems possible, in almost all cases the species effect is considerably larger. In order to demonstrate the biological context of these results, significantly upregulated genes were superimposed onto the rat KEGG’s pathways^52^,^53^: DNA replication (Fig. 2), and Fanconi Anemia (Fig. 3), which are both significantly enriched among *Spalax* upregulated genes. Overall, genes involved in this set of functions act together and protect from DNA-damage, and replication stress. In the context of *Spalax* adaptive evolution, these genes may protect against damage induced during hypoxia-reoxygenation cycles.

The second supergroup (Table 1), which can be defined as the chromosomal segregation group, includes functional groups associated with the kinetochore protein structure, homologous chromosomal segregation, and cell cycle. Specifically, among kinetochore genes upregulated at >4 fold in *Spalax*, we find that all the four genes from which the Ndc80 complex is composed of are upregulated in *Spalax (NUF2, SPC24, NDC80*, and *SPC25*), a complex which is critical in the M phase checkpointing^54^. Overall, the observed overrepresentation of the chromosomal segregation supregroup may be associated with differences in the control and checkpointing of the Metaphase between *Spalax* and rat, specifically (although not exclusively) at the kinetochore level.

The third subgroup (Table 1), which can be defined as the extracellular matrix (ECM) group, includes genes whose products are bound to the ECM, or mediate ECM-receptor interactions (Supplementary Fig. S3). In a previous RNA-Seq *Spalax* brain microarray study^22^, these ECM related terms were significantly overrepresented among hypoxia responsive genes, and were part of a cluster of functionally-related terms associated with hypoxia-induced angiogenesis.

As shown in Table 1 and Table 2, similar GO enrichment patterns are seen using two different foreground-group selections, namely: using significantly upregulated genes with log_2_FC >1 as in Table 1, or using all the significantly upregulated genes as shown in Table 2. In the second analysis (Table 2) we identified the GO terms “chromolomal maintainance”, and specifically “MCM complex” (minichromosome maintenance protein complex), which is required for the formation and elongation of the replication fork. For illustrative purpose, Figures S5 and S6 show differentially expressed genes superimposed on the BER and cell-cycle pathways.

Among the down-regulated genes with atleast two fold change (Table 1), several groups of genes are enriched, which include genes associated with extracellular vesicles (specifically the lysosome). We further searched for functional enrichment among all significantly downregulated genes, including genes that exhibit small significant changes (less than two fold decrease in *Spalax*). This allowed detecting multiple functionally enriched terms, encompassing hundreds of DE genes (Table 2). We grouped these functional terms into two major clusters: (1) unfolded protein binding and their breakdown; (2) carbohydrate metabolism and cellular respiration. The latter cluster includes the functional group NADH dehydrogenase complex I, which refers to an NADH oxidation stage that initiates the electron transport chain of the oxidative phosphorylation pathway (e.g., *NDUFB5/7/9/10, NDUFS1/4, NDUFV1/2/3, NDUFC2,* and *NDUFA9*; Supplementary Fig. S7 illustrates DE genes related to oxidative phosphorylation pathway). In addition, downregulated genes belonging to the significantly enriched term Parkinson’s disease (Table 2, rat KEGG 05012), are part of the oxidative phosphorylation pathway. Furthermore, genes associated with the hexokinase enzyme activity, a key enzyme of the first stage of the glycolysis, are significantly enriched among *Spalax* downregulated genes.

### Validation of RNA-Seq results by absolute qRT-PCR quantification

**Figure 4:**
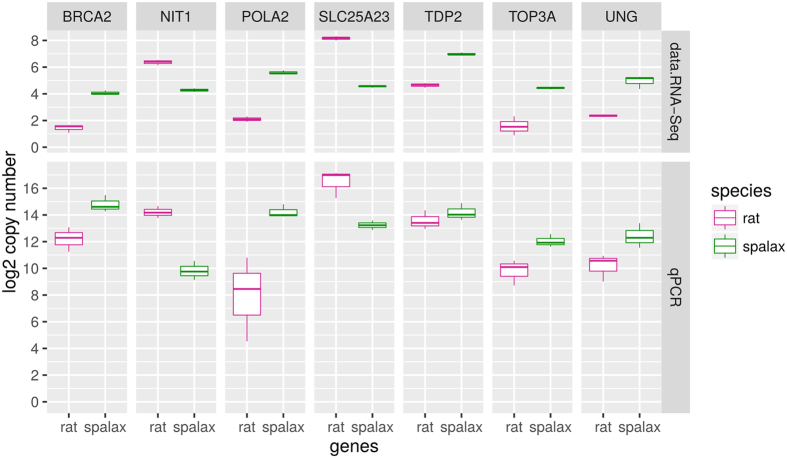
Comparison of RNA-Seq normalized read counts, to the normalized Real time Quantitative PCR copy numbers. The upper panel shows the RNA-Seq read normalized counts (log_2_FPKM) for seven genes, five upregulated in *Spalax* vs. rat in the brain, and two downregulated. The lower panel shows log_2_ normalized Real time Quantitative PCR copy numbers for the same genes, in *Spalax* vs. rat brain samples. In six out of seven tests, the results of both methods show the same trend, with significant DE. Figure S4 shows the same comparison against total qPCR copy numbers, rather than normalized copy numbers.

For qRT-PCR, we selected seven genes of interest, among them five that are up-regulated in *Spalax (POLA2, BRCA2, TDP2, TOP3A*, and *UNG*) and two that are down-regulated (*NIT1, SLC25A23*) in *Spalax*, compared to rat. The selected up-regulated genes are known to play important roles in DNA metabolism and repair, while the down-regulated genes are associated with apoptotic pathways in the mitochondrion. The evaluated qRT-PCR transcripts levels of the genes *POLA2, BRCA2, TOP3A, NIT, and SLC25 A23* showed similar fold-change trends, and were significantly differentially expressed (*p* < 0.05, Welch’s t-test) (Fig. 4, Supplementary Fig. S4). The gene *UNG* gave a marginally significant estimation *(p* = *0.07,* Welch’s t-test). This clearly stems from a large biological variance between samples, which is seen in both the qRT-PCR and the RNA-Seq estimations, due to the same single *Spalax* outlier call in a specific individual. Thus, the test of the gene *UNG* can clearly be considered as a successful validation of the RNA-Seq analysis. The qRT-PCR DE results for the gene TDP2 were not significant. Overall, according to the Sign Test, the probability of the observed distribution of successes by chance is *p* = 0.0625 (assuming we expect a proportion of 50% correct DE calls by chance), indicating that the qRT-PCR results corroborate with the RNA-Seq results.

## Discussion

Here we present a comparison of transcript abundance in *Spalax* vs. rat whole brain tissues. Our analysis revealed that genes up-regulated in the *Spalax* brain, compared to the rat brain, are significantly associated with DNA repair, DNA metabolism, DNA recombination, cellular response to DNA damage stimulus, cell cycle, chromosomal segregation, ECM receptor-ligand interactions, among other functions. Significantly down-regulated *Spalax* genes were associated with unfolded protein binding and their breakdown, carbohydrate metabolism and cellular respiration, and extracellular vesicles activity (see Table 1, Table 2, and Supplementary Table S3). We further studied the biological context of these findings based on the literature, as detailed below.

We find that *Spalax* down-regulated genes are significantly associated with carbohydrate metabolism, lipid metabolism, redox metabolism, mitochondria inner membrane activity, and oxidative phosphorylation (see Table 2, Supplementary Table S3). Cellular respiration, via oxidative phosphorylation, is a major source of ROS^55^ that may lead to the accumulation of DNA damage^56^. Therefore, these results seem to reflect differences in the regulation of glucose, ATP, and ROS levels between *Spalax* and rat. ROS signaling influences the cellular control of the cell growth and development, cell survival, immune response, angiogenesis, tumorigenesis, cell cycle, and the antioxidant and DNA repair responses^56^,^57^,^58^. In addition, tight control of ROS and ATP production can be critical during hypoxia in *Spalax*, where baseline levels of ROS and ATP are extremely disrupted. These results can also reflect interspecies differences in the control of aging, since excessive ROS production, for example as a consequence of hypoxia, was suggested to lead to aging. Specifically, studies show that while stem-cell differentiation and renewal require moderate ROS production, ROS excess facilitates stem cells exhaustion and aging^56^,^58^,^59^.

**Figure 5:**
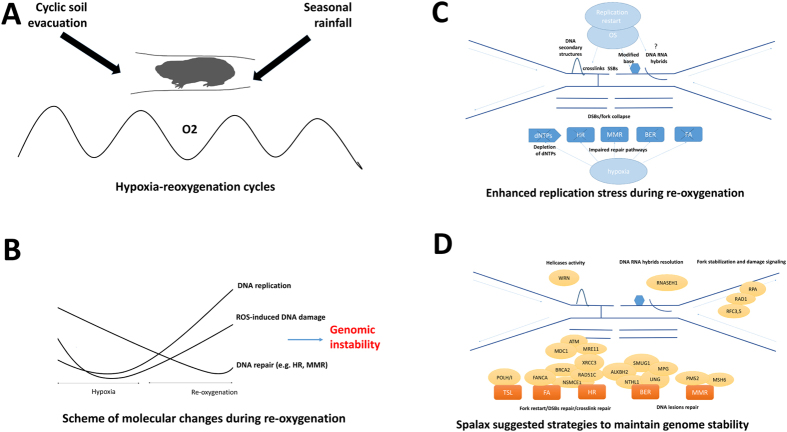
Suggested model of genome stability maintenance in *Spalax*. (**A**) *Spalax* endures oxygen fluctuations in subterranean environments induce oxidative-stress; **(B)** Under hypoxia, dNTPs are depleted, replication is arrested, and transcript abundance of genes in DNA-repair pathways is halted. Reoxygenation after short-term hypoxia leads to replication restart and is accompanied by oxidative stress, which induces additional DNA damage. Yet, compared to murines, *Spalax* shows a higher normoxic transcript abundance of repair and DNA-metabolic genes, which may allow better responses to hypoxia-reoxygenation; **(C)** Depleted dNTPs and additional DNA damage lead to stalled/collapsed replication fork and enhanced replication stress at the same time when DNA repair pathways have not yet recovered; **(D)** Putative protective mechanisms in *Spalax* cells may include counteracting a shortage of repair genes and replication factors by up-regulating repair pathways (e.g., HR, FA, and MMR), oxidative damage repair (e.g., BER), stabilizing of stalled forks, and restarting stalled/collapsed forks.

In its natural habitat, *Spalax* is exposed to cyclic events of hypoxia, followed by reoxygenation (Fig. 5A). In aboveground mammals, cellular-hypoxia induces abrogation of DNA-replication, and transcriptional downregulation of DNA-repair genes^14^. Following reoxygenation, DNA replication recovery was found to precede DNA repair recovery^18^ (Fig. 5B). Consequently, at reoxygenation onset and upon replication restart, impaired coordination between the replication and the repair machinery in the presence of ROS-induced DNA damage can enhance replication stress (Fig. 5C) and lead to genome instability in replicative tissues, and cause accumulation of DNA damage in non-replicative tissues. Our results indicate that, as compared to an aboveground murine, *Spalax* maintains significantly higher normoxic transcript levels of DNA-damage repair and DNA metabolic genes (Tables 1 and 2, Fig. 2, and Fig. 3). We suggest that high-baseline transcript levels of repair and replication genes protect *Spalax* against hypoxia-induced reduction of DNA-repair and DNA damage upon reoxygenation (Fig. 5D). This mechanism can therefore prevent defective and uncoordinated resumption of replication and genomic instability progression at the reoxygenation onset.

In support of this interpretation, we find that many of those DNA metabolic genes, and DNA repair genes, that are upregulated in *Spalax*, were previously found to be repressed under hypoxia in other organisms, and/or to be associated with the protection against replication- and oxidative- stress (Table 3). Genes that show higher levels of transcript abundance in *Spalax* are involved in the initiation and elongation phases of replication (e.g., *POLA1/2* and *RFC3/5*, respectively, see Table 3 and Fig. 2); both phases are abrogated under hypoxia in other species^14^. The observed upregulation of these factors in *Spalax* may play a role in ensuring proper replication-restart during reoxygenation. In addition, genes related to the repair of errors during DNA replication via the mismatch repair (MMR) pathway are also upregulated in *Spalax*. For example, the gene *MSH6* which is downregulated under hypoxic conditions in other species and whose downregulation is correlated with an increase in genomic instability^60^. Notably, *Msh6* functions in a complex with *Msh2*, a gene whose mRNA levels remains stable following hypoxia in *Spalax*, as opposed to ~2 -fold decrease observed in mice^61^. Among the upregulated genes, we have also identified genes responsible for the removal of factors that may stall the replication fork. For example, *RNASEH1*, which is involved in the removal of DNA:RNA hybrids^62^,^63^, was found to be upregulated in *Spalax* in the current study.

Other genes that are upregulated in *Spalax* are involved in base excision repair (BER) pathway (e.g., *SMUG1, UNG*, and *MPG*). This pathway is critical in the repair of oxidative DNA damage^64^, and is also repressed under hypoxia^65^ in other species. Noteworthy, during the intermediate stages of the BER process itself, additional DNA lesions (e.g., single-strand break, SSBs) are created^66^. Thus, when BER is activated as a response to oxidative damage under replication-restart following reoxygenation, the risk for stalled forks and fork collapse may increase as the complementary DNA repair factors are not yet recovered following hypoxia; all together this may lead to a “snowball” effect and eventually to genomic instability deterioration. Hence, an enhanced activity to stabilize and restart the fork or to repair double-strand breaks (DSBs) in case of fork collapse may be required in *Spalax*; Indeed, as found in the current study, genes related to the stabilization of stalled replication forks and to fork restart (e.g., *RAD1*, which is part of the 9-1-1 complex) and genes related to DSB sensing and repair (e.g., *ATM, MRE11, RAD51C*, and *BRCA2*) are also upregulated in *Spalax*. The latter set of genes are involved in HR repair pathway, which is also known to be suppressed under hypoxia in other species^18^. For example, in other mammals, the downregulation of *RAD51* and *BRCA1* under hypoxia, both at protein and mRNA levels, persisted for up to 48 hours after cell reoxygenation and accompanied by decreased HR capability^18^.

An additional subgroup of genes, upregulated in *Spalax*, participates in the FA pathway, a critical pathway for stabilizing stalled replication forks and prevent their degradation^67^ (see Fig. 3). This pathway coordinates HR, nucleolytic incision, and translational synthesis (TLS) to remove interstrand cross links (ICLs)^68^, which are a bidirectional blockage of the replication fork. *Spalax* upregulates genes that are involved in this pathway (e.g., *FANCA*), HR factors as mentioned above, and genes related to TLS (e.g., *POLH, USP1*)^69^,^70^, which are downregulated under hypoxia^67^ in other species. Other interesting genes that were found to be upregulated in *Spalax* include (1) the *WRN* gene, which encodes for a multifunctional helicase involved in DSBs repair^71^, recovery of stalled replication forks^72^, and resolution of repetitive DNA elements and DNA secondary structures that may stall replication^17^; (2) RPA interacting protein (*RPAIN*) gene, which is involved in the import of replication protein A (RPA) complex into the nucleus. RPA is a critical protein in repair and replication, which stabilizes single-stranded DNA (ssDNA) intermediates and prevents re-annealing of complementary DNA. It is involved in the stabilization of stalled forks and in the recruitment and activation of other DNA damage response (DDR) proteins. When RPA is depleted, the replication fork collapses^15^ and DSBs may occur^16^,^63^. Therefore, enhanced import of RPA to the nucleus may serve *Spalax* under the above-mentioned threats; (3) genes involved in telomere maintenance, for example, *MRE11A, RNASEH1*^73^,^74^ and the gene *TELO2*, which is associated with cellular resistance to DNA damage stresses and telomere length regulation; (4) genes involved in chromosomal segregation. Defects in chromosomal segregation were shown to promote cancer^75^ and aging^76^. The upregulation of these genes may serve *Spalax* as an additional mechanism to avoid genomic instability, in addition to the enhancement of replication and repair factors during DNA synthesis. The involvement of cell cycle factors related to kinetochore may indicate a more controlled G2/M checkpoint that further prevents from cells with unrepaired damage to progress into mitosis. We conclude that the involvement of genes related to genome maintenance, and DNA repair and replication, protects *Spalax* against damage induced by oxygen fluctuations, and may also be associated with its longevity and cancer resistance^77^.

## Additional Information

**How to cite this article**: Malik, A. *et al*. Genome maintenance and bioenergetics of the long-lived hypoxia-tolerant and cancer-resistant blind mole rat, *Spalax*: a cross-species analysis of brain transcriptome. *Sci. Rep.* **6**, 38624; doi: 10.1038/srep38624 (2016).

**Publisher's note:** Springer Nature remains neutral with regard to jurisdictional claims in published maps and institutional affiliations.

## Supplementary Material

Supplementary Figures and Table S4

Supplementary Dataset 1

Supplementary Dataset 2

Supplementary Dataset 3

Supplementary Dataset 5

## Figures and Tables

**Table 1:** Functionally enriched terms among genes significantly up/down regulated in *Spalax* vs. rat, in the brain, at above two fold change.

Category	Functional terms	Adjusted *p* values				
		CP	GP	GS	GR	DE
Log_2_FC > 1.0 Adj. *p* < 0.05 Spalax > rat brain	(I) DNA metabolic process	5.7E-07	2.3E-04	2.6E-04	2.6E-05	102
	(I) DNA replication/DNA-dependent DNA replication	2.7E-03	9.6E-03	1.3E-02	8.6E-03	23
	(I) DNA repair	1.4E-03	4.1E-03	2.0E-02	1.7E-02	59
	(I) Fanconi Anemia pathway (KEGG)	1.1E-02	6.3E-02			13
	(I) cellular response to DNA damage stimulus	1.4E-03		9.4E-02	*2.1E-01*	79
	(I) DNA recombination/recombinational repair	7.8E-03	5.7E-03			29
	(II) homologous chromosome segregation	7.8E-03	2.2E-02	9.4E-02	*4.4E-01*	11
	(II) kinetochore/condensed chromosome kinetochore	5.7E-03	5.0E-02	2.0E-02	1.4E-02	22
	(II) chromosomal region/centromeric region	5.7E-03	7.9E-03	9.4E-02	1.4E-02	38
	(II) Cell cycle/Mitotic Metaphase		8.1E-02	9.5E-02	4.6E-02	88
	(III) extracellular matrix/proteinaceous extracellular matrix	2.9E-05	3.2E-02	3.6E-02	1.9E-09	56
	(III) heparin binding	4.7E-03				20
	(III) ECM-receptor interaction	1.6E-02		9.5E-02		17
Log_2_FC < 1.0 Adj. *p* < 0.05 Spalax < rat brain	(IV) extracellular space	5.9E-09	1.9E-06	2.4E-03	1.4E-05	119
	(IV) extracellular vesicle/lysosome/exosome	8.7E-03	1.9E-06	1.5E-02	2.7E-03	254
	(V) G-protein coupled receptor activity/G-protein complex	8.1E-03		5.4E-02	6.8E-02	75

Category column: (1) genes significantly up-regulated in *Spalax* vs. rat, in the brain, with at least two fold increase (log_2_FC > 1.0); (2) genes down-regulated, with least two fold decrease (log_2_FC < −1.0); Terms column: groups of functionally-related terms are marked in Roman numerals; Adjusted p values heatmap: FDR-corrected *p* values reported by the programs cluster-profiler (CP), g-profiler (GP), goseq (GS), and Gorilla (GR). The total count of DE genes is shown in the last column (DE). Note that Gorilla tool does not include a KEGG/Reactome databases, and terms that were not tested, or were not found to be enriched by specific tools, are shown as blank cells. Significant p-values are underlined, and p-values > 10^−1^ in italics.

**Table 2:** Functionally enriched terms among all significantly up/down regulated in *Spalax* vs. rat, in the brain.

Category	Term (LR-Path RNA-enrich)	adj. p value	Background	DE
Significantly upregulated (spalax > rat)	(I) DNA Replication	1.3E-02	70	48
	(I) DNA metabolic process	1.0E-02	647	273
	(I) DNA Repair	2.2E-02	98	76
	(I) DNA Damage	3.8E-02	100	79
	(I) MCM complex	1.6E-02	8	6
	(II) condensed chromosome kinetochore	9.3E-03	9	8
	(II) nuclear chromosome	9.3E-03	227	126
	(II) condensed chromosome, centromeric region	1.9E-02	13	11
	(II) nuclear chromosome part	3.7E-02	209	116
	(II) Centromere	1.4E-02	24	18
	(II) Chromosome Maintenance	3.1E-02	27	22
	(II) CDK Regulation of DNA Replication	2.8E-02	9	7
	(III) heme-copper terminal oxidase activity	8.5E-03	5	3
Significantly downregulated (spalax < rat)	(IV) Ubiquitin proteasome pathway	3.8E-02	27	16
	(IV) HECT-domain (ubiquitin-transferase)	1.8E-02	17	8
	(IV) Ubiquitins	1.8E-02	77	48
	(IV) unfolded protein binding	8.5E-03	38	27
	(IV) proteasome regulatory particle	7.7E-03	12	8
	(V) NADH dehydrogenase complex	1.9E-02	21	11
	(V) mitochondrial respiratory chain	2.4E-02	22	12
	(V) mitochondrial inner membrane	2.4E-02	200	111
	(V) respiratory chain	2.4E-02	40	13
	(V) NADH dehydrogenase activity	1.7E-02	15	8
	(V) Hexokinase	5.0E-03	6	4
	(V) Parkinson’s disease	1.0E-03	43	28
	(VI) anion channel activity	2.8E-02	38	19
	(VI) sodium channel regulator activity	8.5E-03	18	9
	(VII) extracellular vesicular exosome	2.9E-03	1142	791

The table shows significantly enriched terms among all significantly up- or down-regulated genes, in Spalax vs. rat, using the tool LR-Path RNA-enrich. This tool corrects for read-counts biases (Methods). In this test all significantly regulated genes were defined as DE (for all fold-change differences); Terms column: groups of functionally-related terms are marked in Roman numerals. Significant p-values are underlined.

**Table 3:** Known functions of DNA-repair and DNA-metabolism genes, DE at higher levels in *Spalax* compared to rat.

Strategy	Pathway	Symbol	Role in the pathway (reference)
Availability of replication factors	replication	RPAIN*	Imports RPA into the nucleus. RPA stabilizes ssDNA and prevents its reannealing, recruits and activates different proteins and complexes involved in DNA metabolism and repair
		POLA1,2	Replication initiation. Part of the DNA polymerase alpha complex^78^
		RFC3,5	Replication elongation. Part of the RFC complex clump loader (load f PCNA onto DNA)^79^
		FEN1	Cleaves the 5-overhanging flap structure that is generated during replication^80^
		POLG	Replication and repair in the mitochondria
Enhanced repair of mismatched bases during replication	MMR	RFC3,5	Part of the RFC complex. Involved in mismatch-provoked excision^79^,^81^
		MSH6	Mismatch recognition complex. MSH2/MSH6 (MutSa) heterodimer^82^
		PMS2	Part of the MutLa which in complex with MutSa and in the presence of RFC and PNCA, introduces single-strand breaks near the mismatch to enable degrading the strand
		WRN	Interacts with RPA and MutLα (MLH1-PMS2) as well as MutSa (MSH2/MSH6) and MutSβ (MSH2/MSH3), which stimulate its helicase activity^83^,^84^
		RPA	Binds nicked DNA before MutSα and MutLα, stimulates mismatch-provoked excision, protects ssDNA gapped region, facilitates DNA re-synthesis, and stimulates WRN^84^,^85^
Stabilizing stalled forks	ATR path.	RAD1	Part of the 9-1-1 complex that associates with TOPBP1 to stimulate ATR kinase activity^86^,^87^
		RPA	stabilizes ssDNA, recruitment of ATRIP to activate ATR kinase
		TOPBP1	Activation of ATR signaling cascade and CHK1 phosphorylation
		TIMELESS	Associates with RPA2 to stabilize stalled forks and to promote the accumulation of CHK1 to RPA-ssDNA regions where it can be activated by ATR^86^
Repair lesions that may stall the replication fork	BER	RAD1	Part of the 9-1-1 complex - a sliding clamp platform on DNA for several proteins involved in long-patch BER (LP-BER). Stimulates POLB, FEN1, and LIG1 activity^87^
		FEN1	Cleaves within the apurinic/apyrimidinic (AP) site-terminated flap. Prevents flaps from equilibrating into structures that lead to duplications and deletions.
		NTHL1	DNA glycosylase activity (substrates containing oxidized pyrimidine) and AP lyase activity^88^
		PNKP	Ensures that DNA termini are compatible with extension and ligation
		SMUG1	DNA glycosylase that removes uracil from ssDNA and ssDNA. initiates BER
		UNG	Eliminating uracil from DNA molecules by cleaving the N-glycosylic bond. initiates BER
		ALKBH2	Repair of alkylated DNA^64^,^89^
		MPG	Resolve alkylation lesions by hydrolysis of deoxyribose N-glycosidic bond
		WRN	Participates in LP- BER by unwinding 5′ flaps and interacting with BER proteins^71^
	RNA:DNA hybrids	RNASEH1	Endonuclease that specifically degrades RNA of RNA-DNA hybrids
		BRCA2	Interacts with the transcription and export complex 2, required to prevent R-loop-associated DNA damage
Collapsed-fork restart, DSBs and ICL repair	ATM path.	MRE11A	Part of the MRN complex. Possesses single-strand endonuclease and double-strand-specific 3-5 exonuclease activities. Required for ATM kinase signaling.
		ATM	DNA damage sensor. Phosphorylates H2AX/H2AFX at DSBs
		MDC1	Interacts with phosphorylated H2AX near sites of DNA DSBs. facilitates recruitment of the ATM and MRE11.
		EYA3	Promote the recruitment of DNA repair complexes containing MDC1.
	HR	MCM8	Part of the MCM8-MCM9 complex, involved in HRR following ICL. Promotes resection of DSB ends by MRN complex and RAD51 recruitment^90^,^91^
		TOP3A	Essential component of the RMI complex that plays an important role in the processing of HR intermediates to limit DNA crossover formation in cells.
		BRCA2	Target RAD51 to displace RPA from ssDNA and stabilize RAD51-ssDNA filaments. Involved in POLH localization at collapsed fork
		RAD51C	Part of BCDX2 and CX3 complexes that bind to the intersection of the four duplex arms of the Holliday junction (HJ) and to junction of replication forks.
		XRCC3	Part of the CX3 complex. Involved in HJ resolution and in processing HR intermediates, downstream RAD51
		NSMCE1	Component of the SMC5-SMC6 complex, involved in DSBs repair by HR and may promote sister chromatid HR by recruiting the SMC1-SMC3 cohesin complex to DSBs.
	FA**	PMS2	Part of MutLa that interacts with FANCJ^92^
		FANCD2	Promotes BRCA2 loading onto damaged chromatin Recruit POLH
		FANCA	Serves as a docking or anchor point at the site of ICL damage for the FA core complex. Interacts with BRCA1^93^
		RUVBL1	Controlling the cellular abundance of FA core complex^94^
	TSL	POLH/I	Incorporating nucleotides opposite a variety of lesions thereby bypasses the lesions^95^
		USP1	Involved in PCNA-mediated TLS by de-ubiquitinating mono-ubiquitinated PCNA

Selected pathways and the role of each gene in the pathway are specified in the table. Information was obtained from a literature survey (unless mentioned otherwise, data was obtained from GeneCards). An extended list of DE genes is provided in Supplementary Table S2. ‘*’ The differentially expressed gene was RPAIN, however, we refer to the function of RPA; ‘**’ in addition to genes calcified under the FA category in the table, additional genes classified under HR and TLS categories in fact participate in the FA pathways as well.
