# Supplementary material for: Genome maintenance and bioenergetics of the long-lived hypoxia-tolerant and cancer-resistant blind mole rat, Spalax: a cross-species analysis of brain transcriptome
Source: Sci Rep. 2016 Dec 9;6:38624. doi: 10.1038/srep38624 (PMC5146665; doi:10.1038/srep38624)
Supplement: Supplementary Figures and Table S4 [file srep38624-s1.pdf]

Genome maintenance and bioenergetics of the long-lived hypoxia-tolerant and cancer-resistant blind mole rat, *Spalax*: a cross-species analysis of brain transcriptome

Assaf Malik,Vered Domankevich, Han Lijuan, Fang Xiaodong, Abraham Korol, Aaron Avivi, Imad Shams

Supplementary Figures

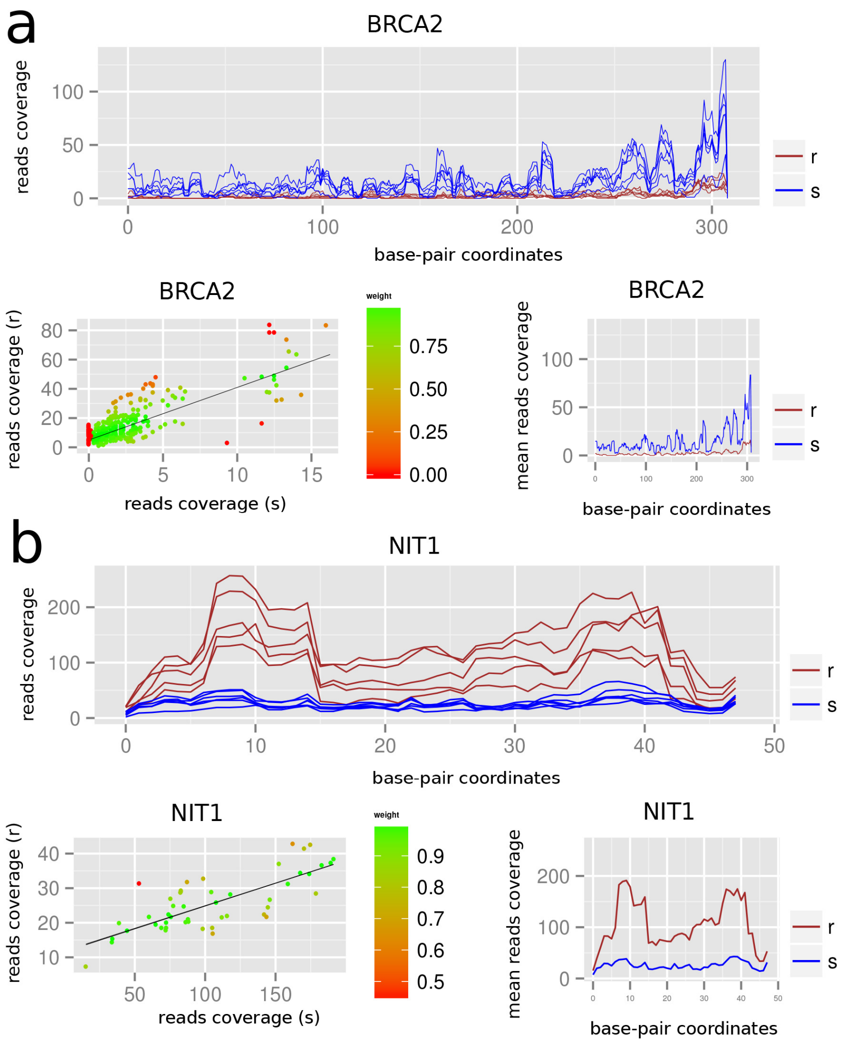

Figure S1: RNA-Seq cross-species coverage levels. Here, the *Spalax* and rat orthologs of the genes *BRCA2* and *Nit1* are used as examples. These genes were tested also in qPCR (Fig. 3, main text). Rat is denoted by "r", and *Spalax* by "s". Main panel (a,b): RNA-seq coverage levels in ~300, and ~50 intervals of 25bp of *Spalax* vs. rat comparable exon regions. Left (a,b): Interspecies coverage relationships. In this case, the Pearson correlation of average coverage between species is ~0.77 and ~0.7, for the two genes, respectively. The green to red weight gradient reflects distance from a regression line found using RLM function. Right (a,b): average coverage levels. In this gene, *Spalax* average coverage is lower than that of rat in most sub-regions, as indicated by a significant Sign-test. For all genes, the DE analysis itself was conducted using EdgeR.

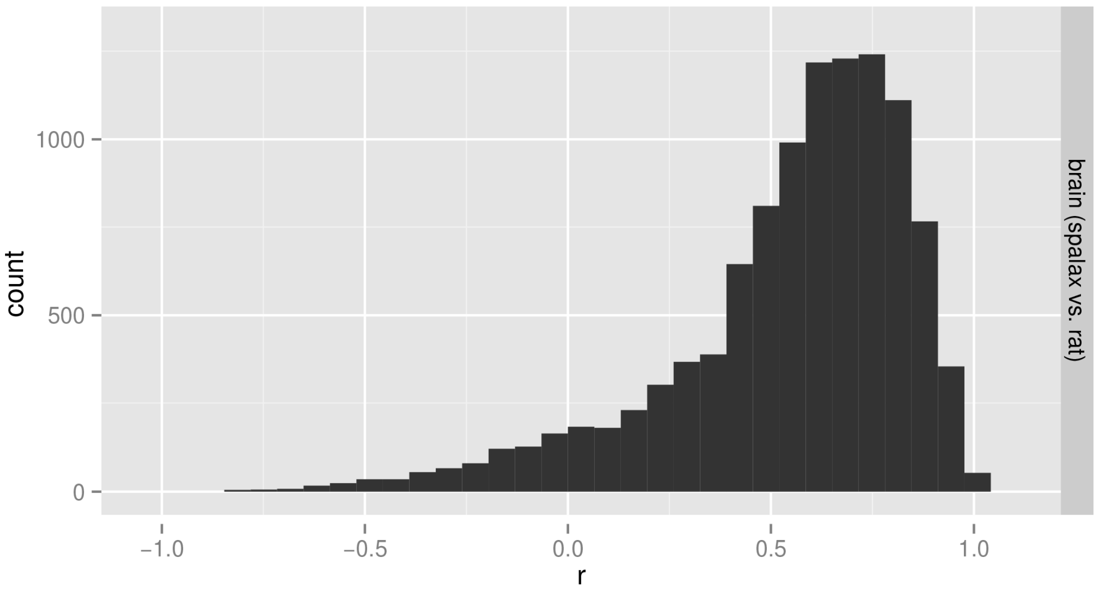

Figure S2: Frequency distribution of Pearson correlations of RNA-Seq coverage, along comparable gene regions of orthologs, based on all tested genes. *Spalax* vs. rat brain transcriptomes were tested. The X axis represents correlation levels. The Y axis represents the counts of genes within each correlation category.

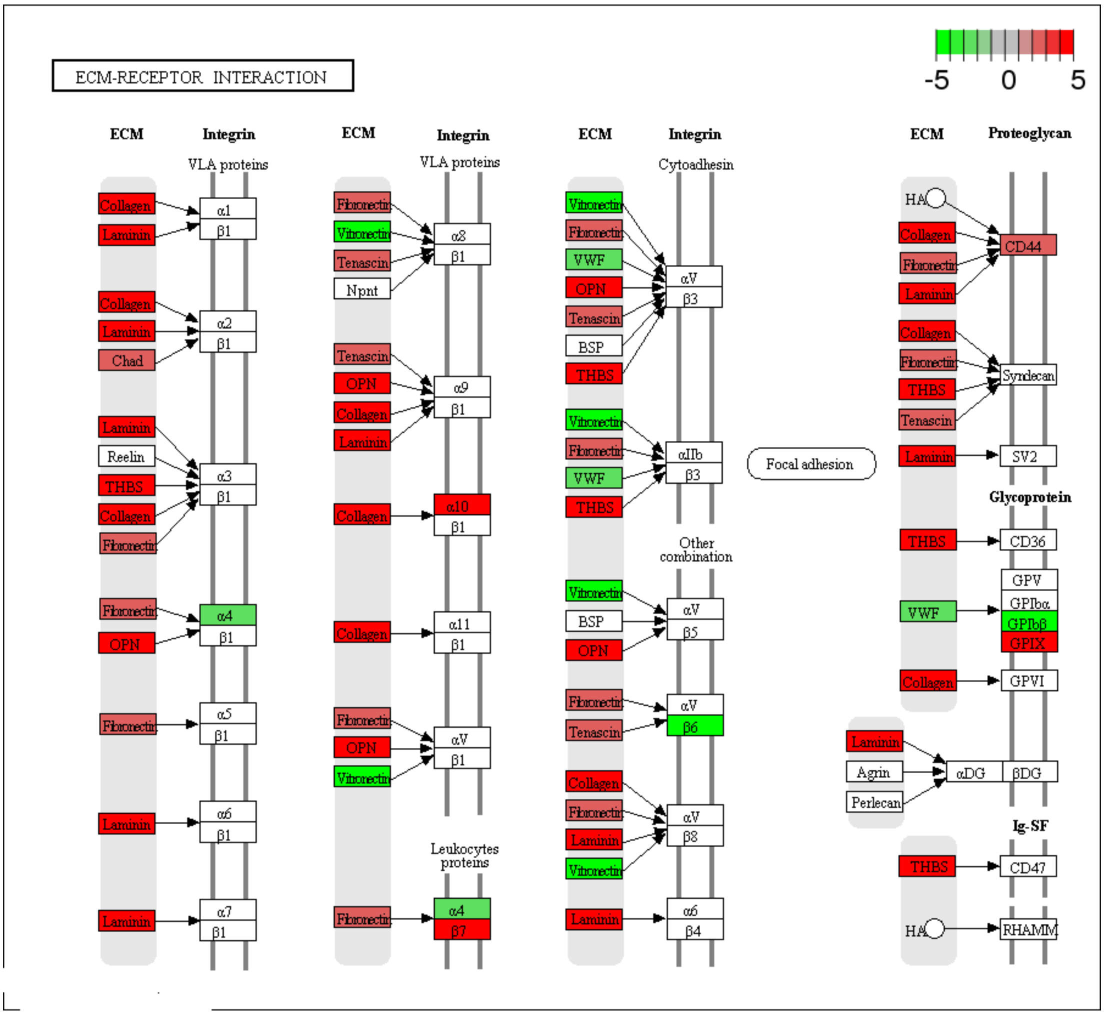

Figure S3: Upregulated/downregulated *Spalax* vs. rat genes, in the brain, within the ECM-receptor interaction group, (fold change > 2 or < -2). DE genes, with at least 2 fold change, in *Spalax* vs. rat are superimposed on the KEGG ECM-receptor interaction pathway of rat (kegg 04512). Color scale represents fold change value.

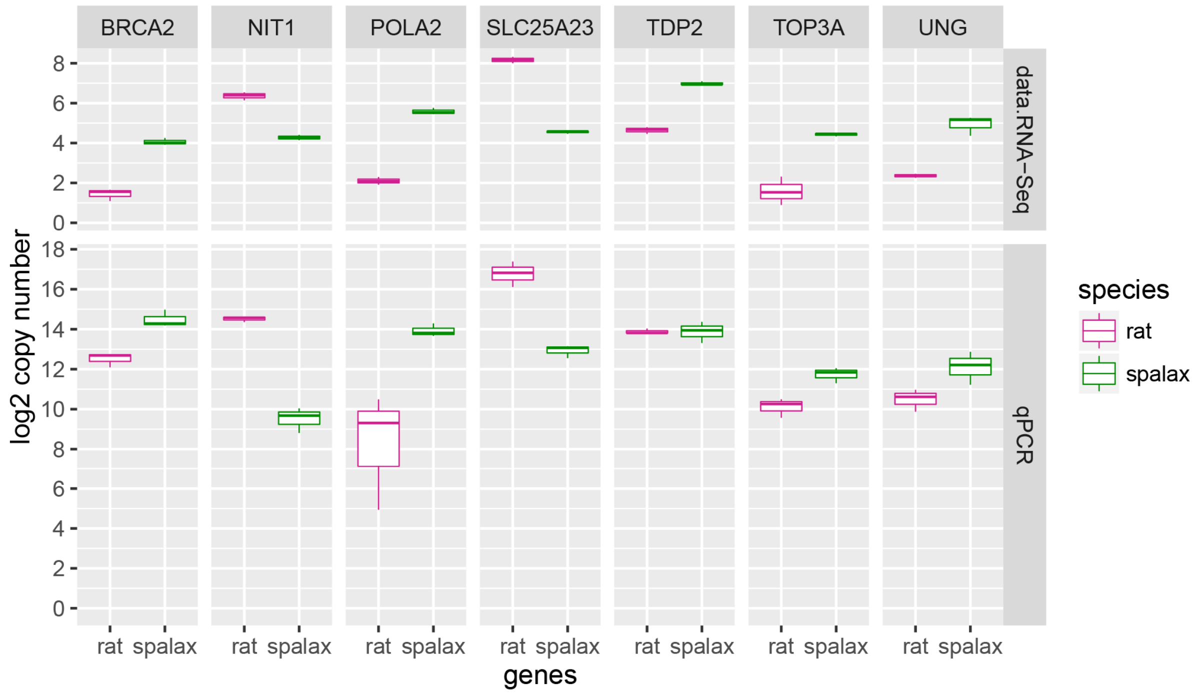

Figure S4: Comparison of RNA-Seq normalized read counts, to the total Real time Quantitative PCR copy numbers. The upper panel shows the RNA-Seq FPKM levels for seven genes, five of them upregulated in *Spalax* vs. rat in the brain, and two downregulated. The lower panel shows to total (not normalized) Real time Quantitative PCR copy numbers for the same genes, in *Spalax* vs. rat brain samples. In six out of seven tests, the results of both tests show the same trend, with significant DE.

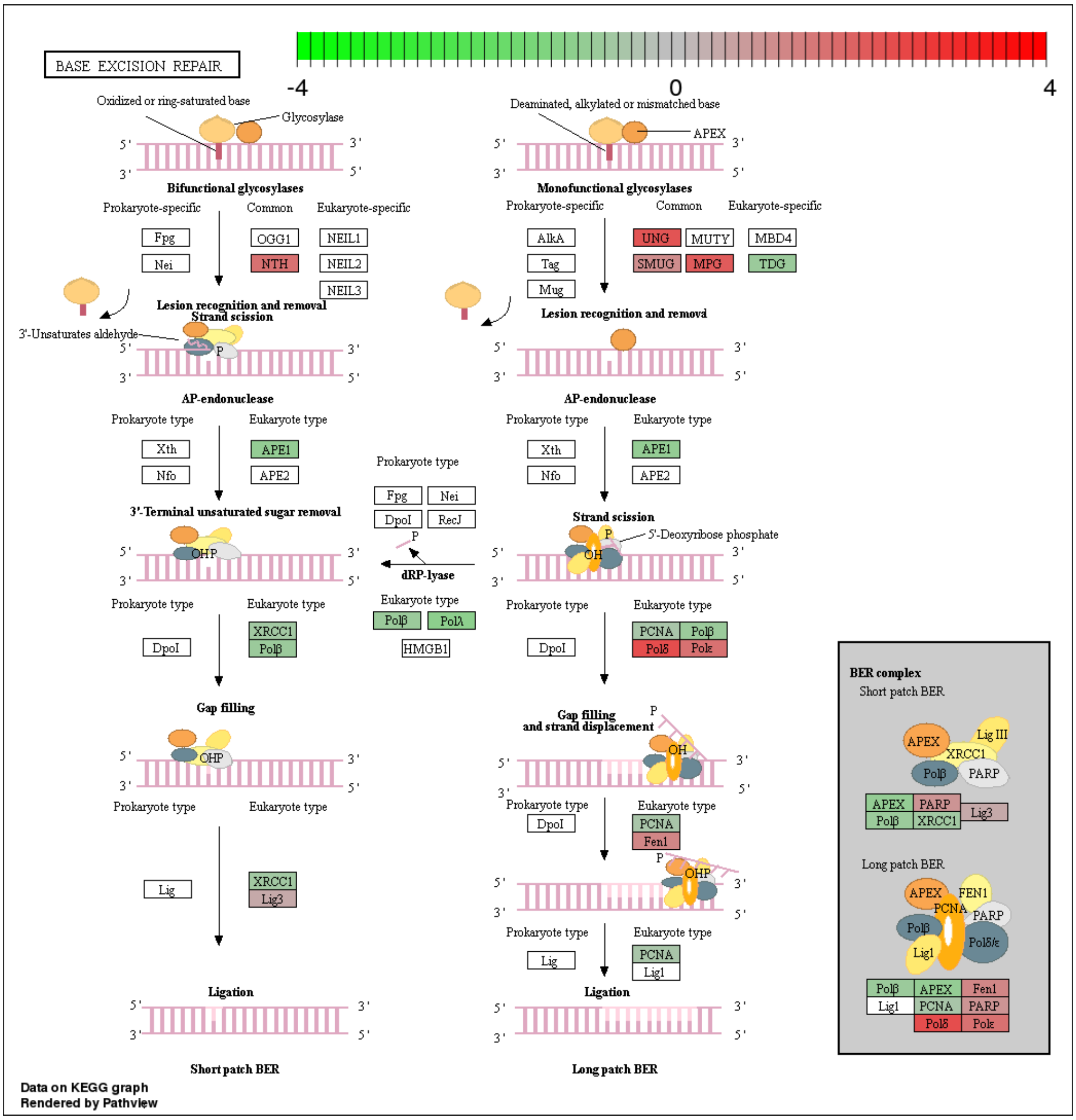

Figure S5: Genes that are significantly differentially expressed, at any fold change, in *Spalax* compared to rat whole brain tissues and are related to Base Excision Repair pathway. Genes with adjusted *p*-value <0.05 (see Table S5) were superimposed on the Base Excision Repair pathway of rat (rno03410, obtained by KEGG, Kanehisa Laboratories). Color scale represents $\log_2$FC value.

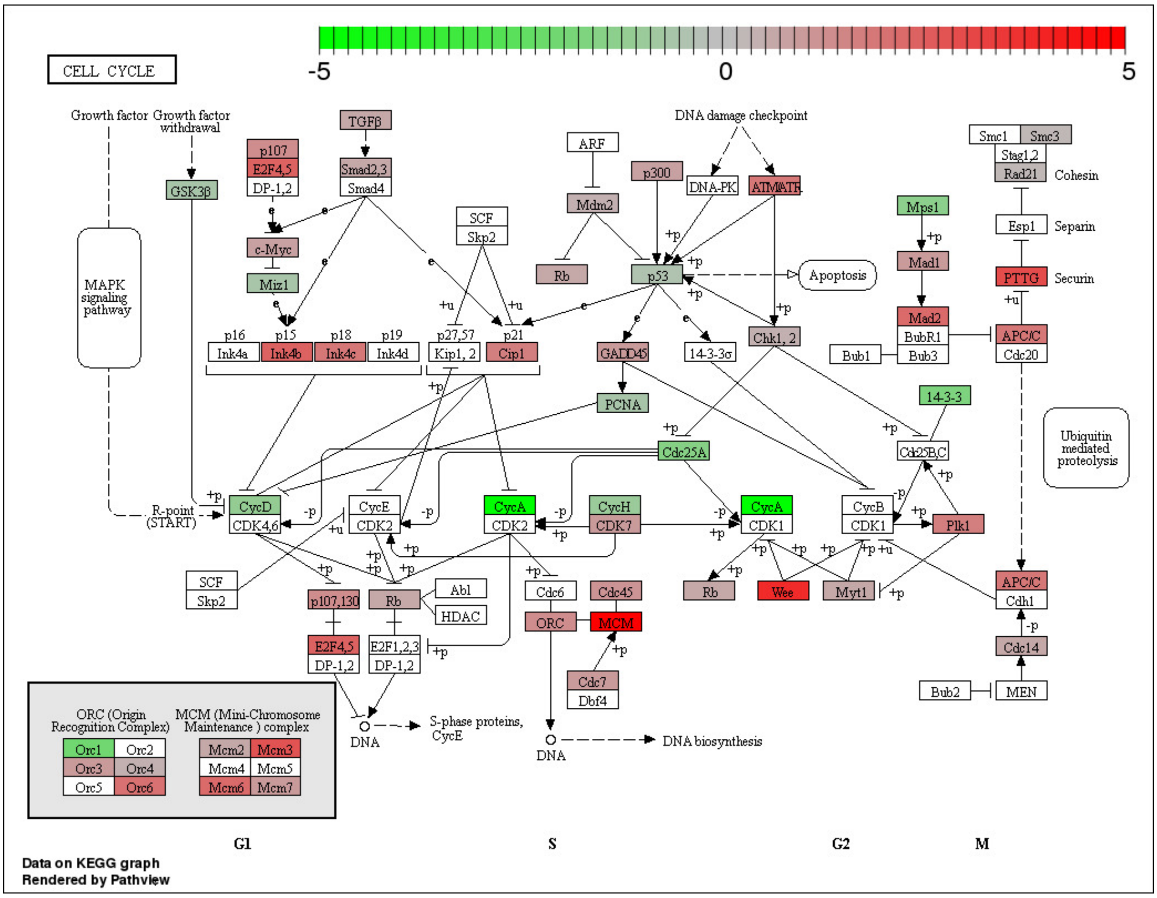

Figure S6: Genes that are significantly differentially expressed, at any fold change, in *Spalax* compared to rat whole brain tissues and are related to Cell Cycle pathway. Genes with adjusted *p*-value <0.05 (see Table S5) are superimposed on the Cell Cycle pathway of rat (rno04110, obtained by KEGG, Kanehisa Laboratories). Color scale represents $\log_2$FC value.

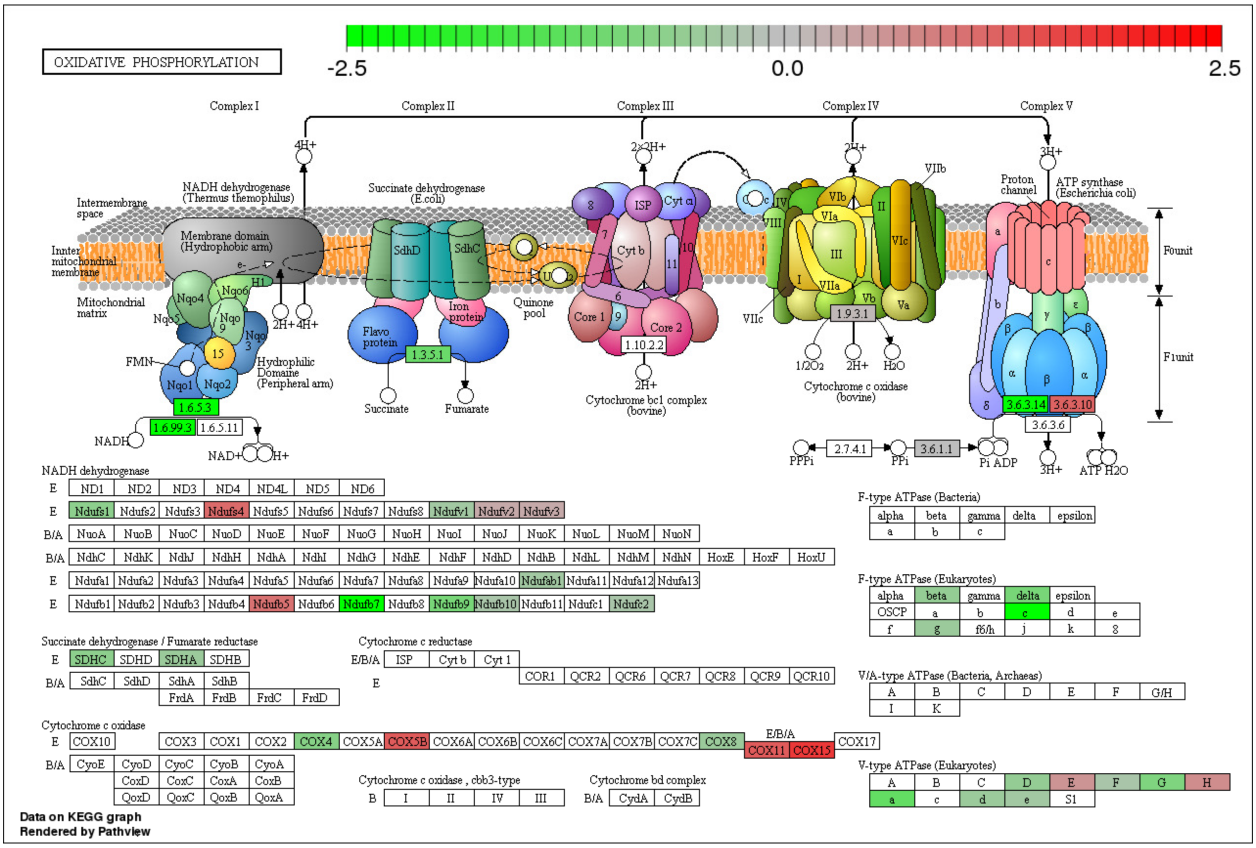

Figure S7: Genes that are significantly differentially expressed, at any fold change, in *Spalax* compared to rat whole brain tissues and are related to Oxidative Phosphorylation pathway. Genes with adjusted p-value <0.05 (see Table S5) are superimposed on the Oxidative Phosphorylation pathway of rat (rno00190, obtained by KEGG, Kanehisa Laboratories). Color scale represents $\log_2$FC value.

Genome maintenance and bioenergetics of the long-lived hypoxia-tolerant and cancer-resistant blind mole rat, *Spalax*: a cross-species analysis of brain transcriptome

Assaf Malik,Vered Domankevich, Han Lijuan, Fang Xiaodong, Abraham Korol, Aaron Avivi, Imad Shams

Table S4: Functional enrichment of differentially expressed genes in *Spalax* vs. rat - brain tissues, using the tool Gorilla. A zip file should be decompressed first. HTML files represent the results of Gorilla, including GO terms hierarchy figures which allow searching for functionally related terms.

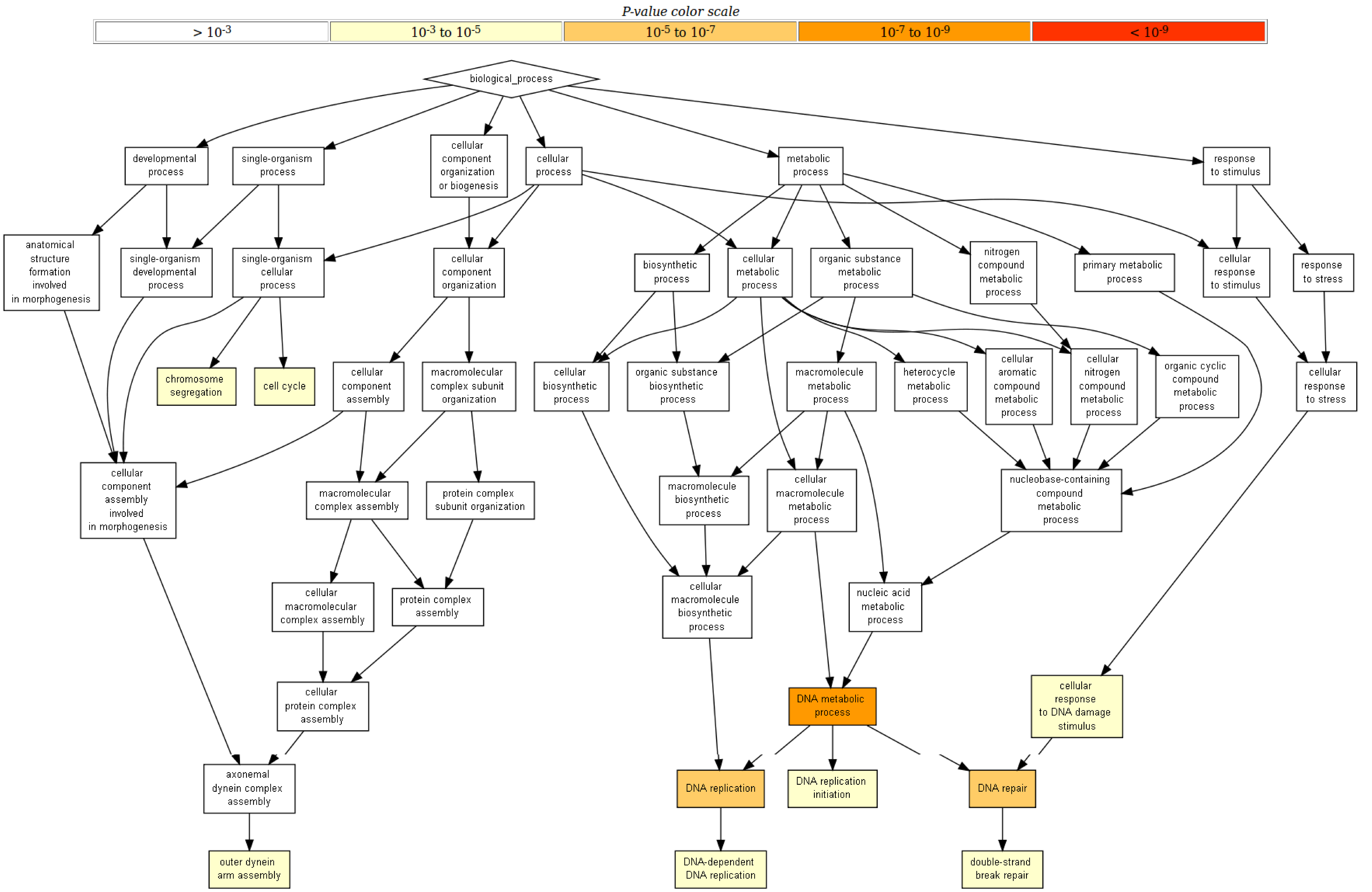

GO term	Description	P-value	FDR q-value	Enrichment (N, B, n, b)	Genes
GO:0006259	DNA metabolic process	2.1E-9	2.58E-5	1.81 (8586,352,1250,93)	[+] Show genes
GO:0006260	DNA replication	1.41E-6	8.64E-3	2.42 (8586,85,1250,30)	[+] Show genes
GO:0006281	DNA repair	4.06E-6	1.66E-2	1.78 (8586,228,1250,59)	[+] Show genes
GO:0007049	cell cycle	1.5E-5	4.61E-2	1.53 (8586,394,1250,88)	[+] Show genes
GO:0006261	DNA-dependent DNA replication	5.14E-5	1.26E-1	3.60 (8586,21,1250,11)	[+] Show genes
GO:0006974	cellular response to DNA damage stimulus	1.03E-4	2.1E-1	1.51 (8586,346,1250,76)	[+] Show genes
GO:0006270	DNA replication initiation	2.61E-4	4.59E-1	3.93 (8586,14,1250,8)	[+] Show genes
GO:0007059	chromosome segregation	2.84E-4	4.37E-1	2.19 (8586,66,1250,21)	[+] Show genes
GO:0036158	outer dynein arm assembly	5.32E-4	7.27E-1	4.58 (8586,9,1250,6)	[+] Show genes
GO:0006302	double-strand break repair	6.26E-4	7.7E-1	2.00 (8586,79,1250,23)	[+] Show genes

Species used: Mus musculus

The system has recognized 9851 genes out of 10359 gene terms entered by the user.
9851 genes were recognized by gene symbol and 0 genes by other gene IDs .
1254 duplicate genes were removed (keeping the highest ranking instance of each gene) leaving a total of 8597 genes.
Only 8586 of these genes are associated with a GO term.

Visualize output in REViGO

The GOrilla database is periodically updated using the GO database and other sources.
The GOrilla database was last updated on Sep 3, 2016

This results page will be available on this site for one month from now (until Oct 3, 2016). You can bookmark this page and come back to it later.

'P-value' is the enrichment p-value computed according to the mHG or HG model. This p-value is not corrected for multiple testing of 12291 GO terms.

'FDR q-value' is the correction of the above p-value for multiple testing using the Benjamini and Hochberg (1995) method.
Namely, for the ith term (ranked according to p-value) the FDR q-value is (p-value * number of GO terms) / i.

Enrichment (N, B, n, b) is defined as follows:
N - is the total number of genes
B - is the total number of genes associated with a specific GO term
n - is the number of genes in the top of the user's input list or in the target set when appropriate
b - is the number of genes in the intersection
Enrichment = (b/n) / (B/N)

Genes: For each GO term you can see the list of associated genes that appear in the optimal top of the list.
Each gene name is specified by gene symbol followed by a short description of the gene

Back to the GOrilla main page

P-value color scale

> 10^{-3}	10^{-3} to 10^{-5}	10^{-5} to 10^{-7}	10^{-7} to 10^{-9}	< 10^{-9}

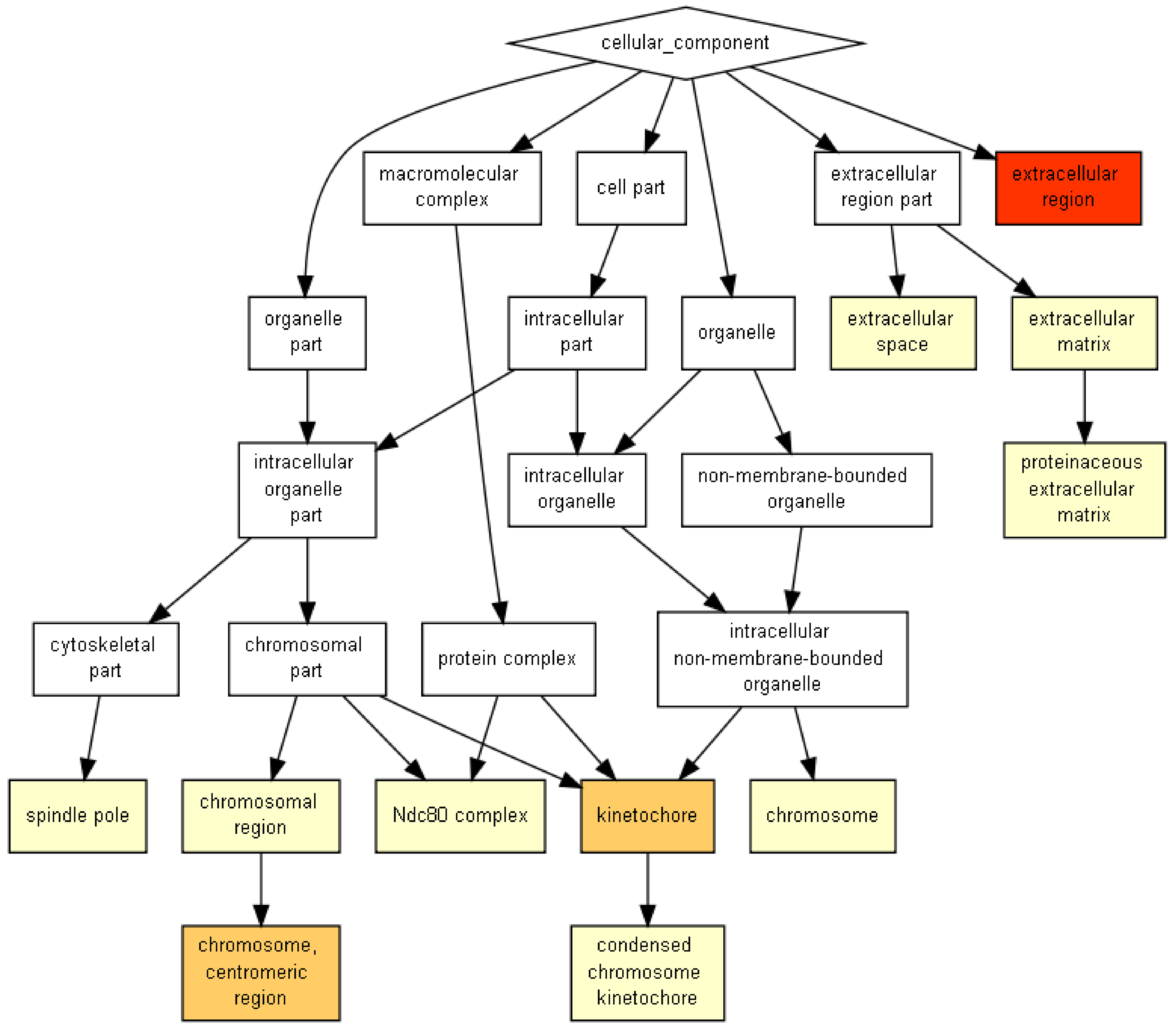

GO term	Description	P-value	FDR q-value	Enrichment (N, B, n, b)	Genes
GO:0005576	extracellular region	8.28E-14	1.26E-10	2.08 (8586,578,741,104)	[+] Show genes
GO:0000775	chromosome, centromeric region	1.44E-6	1.09E-3	2.96 (8586,90,741,23)	[+] Show genes
GO:0000776	kinetochore	2.57E-6	1.3E-3	3.04 (8586,80,741,21)	[+] Show genes
GO:0031262	Ndc80 complex	5.51E-5	2.09E-2	11.59 (8586,4,741,4)	[+] Show genes
GO:0005578	proteinaceous extracellular matrix	5.65E-5	1.72E-2	2.15 (8586,156,741,29)	[+] Show genes
GO:0000777	condensed chromosome kinetochore	6.28E-5	1.59E-2	6.95 (8586,10,741,6)	[+] Show genes
GO:0031012	extracellular matrix	1.39E-4	3.01E-2	1.85 (8586,238,741,38)	[+] Show genes
GO:0098687	chromosomal region	2.2E-4	4.18E-2	2.00 (8586,168,741,29)	[+] Show genes
GO:0005615	extracellular space	3.82E-4	6.46E-2	1.53 (8586,478,741,63)	[+] Show genes
GO:0000922	spindle pole	5.25E-4	7.99E-2	2.79 (8586,54,741,13)	[+] Show genes
GO:0005694	chromosome	8.35E-4	1.16E-1	1.77 (8586,216,741,33)	[+] Show genes

Species used: Mus musculus

The system has recognized 9342 genes out of 9821 gene terms entered by the user.
9342 genes were recognized by gene symbol and 0 genes by other gene IDs .
745 duplicate genes were removed (keeping the highest ranking instance of each gene) leaving a total of 8597 genes.
Only 8586 of these genes are associated with a GO term.

The GOrilla database is periodically updated using the GO database and other sources.
The GOrilla database was last updated on Sep 3, 2016

This results page will be available on this site for one month from now (until Oct 3, 2016). You can bookmark this page and come back to it later.

'P-value' is the enrichment p-value computed according to the mHG or HG model. This p-value is not corrected for multiple testing of 1521 GO terms.

'FDR q-value' is the correction of the above p-value for multiple testing using the Benjamini and Hochberg (1995) method.
Namely, for the ith term (ranked according to p-value) the FDR q-value is (p-value * number of GO terms) / i.

Enrichment (N, B, n, b) is defined as follows:
N - is the total number of genes
B - is the total number of genes associated with a specific GO term
n - is the number of genes in the top of the user's input list or in the target set when appropriate
b - is the number of genes in the intersection
Enrichment = (b/n) / (B/N)

Genes: For each GO term you can see the list of associated genes that appear in the optimal top of the list.
Each gene name is specified by gene symbol followed by a short description of the gene

Back to the GOrilla main page

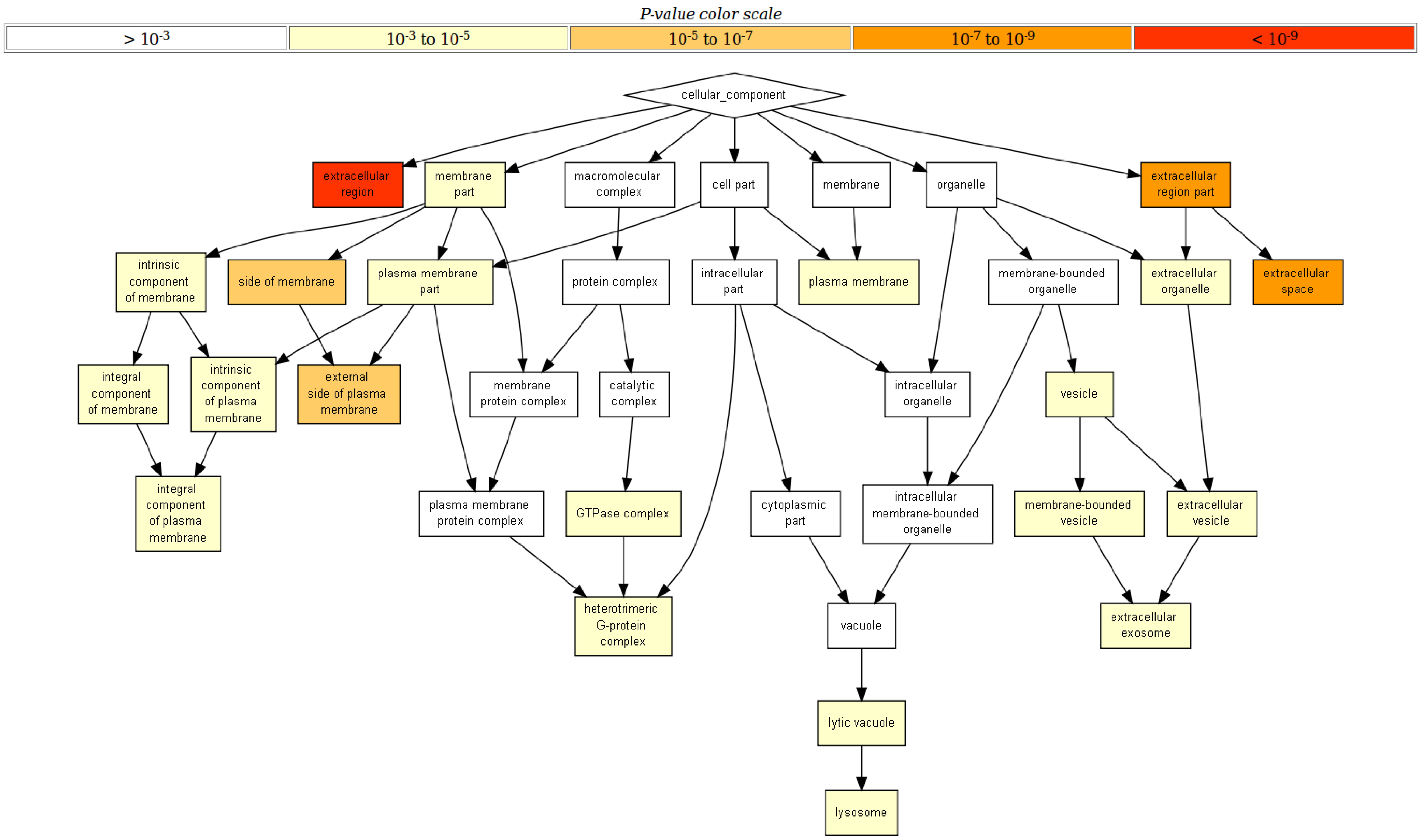

GO term	Description	P-value	FDR q-value	Enrichment (N, B, n, b)	Genes
GO:0005576	extracellular region	2.23E-12	3.38E-9	1.74 (8586,578,1220,143)	[+] Show genes
GO:0005615	extracellular space	1.89E-8	1.43E-5	1.65 (8586,478,1220,112)	[+] Show genes
GO:0044421	extracellular region part	3.81E-8	1.93E-5	1.29 (8586,1737,1220,319)	[+] Show genes
GO:0009897	external side of plasma membrane	6.29E-7	2.39E-4	2.39 (8586,97,1220,33)	[+] Show genes
GO:0098552	side of membrane	6.55E-6	1.99E-3	2.03 (8586,135,1220,39)	[+] Show genes
GO:0043230	extracellular organelle	1.03E-5	2.61E-3	1.27 (8586,1345,1220,243)	[+] Show genes
GO:0031224	intrinsic component of membrane	1.3E-5	2.83E-3	1.20 (8586,2131,1220,363)	[+] Show genes
GO:1903561	extracellular vesicle	1.39E-5	2.65E-3	1.27 (8586,1344,1220,242)	[+] Show genes
GO:0070062	extracellular exosome	1.81E-5	3.07E-3	1.26 (8586,1336,1220,240)	[+] Show genes
GO:0044425	membrane part	2.32E-5	3.53E-3	1.16 (8586,2768,1220,456)	[+] Show genes
GO:0016021	integral component of membrane	3.46E-5	4.79E-3	1.19 (8586,2073,1220,351)	[+] Show genes
GO:0000323	lytic vacuole	4.36E-5	5.53E-3	1.76 (8586,192,1220,48)	[+] Show genes
GO:0005764	lysosome	4.36E-5	5.11E-3	1.76 (8586,192,1220,48)	[+] Show genes
GO:0031226	intrinsic component of plasma membrane	7.78E-5	8.45E-3	1.43 (8586,503,1220,102)	[+] Show genes
GO:0044459	plasma membrane part	8.54E-5	8.66E-3	1.27 (8586,1122,1220,202)	[+] Show genes
GO:0005887	integral component of plasma membrane	1.68E-4	1.6E-2	1.42 (8586,471,1220,95)	[+] Show genes
GO:0031988	membrane-bounded vesicle	1.96E-4	1.76E-2	1.20 (8586,1611,1220,275)	[+] Show genes
GO:0005886	plasma membrane	4.29E-4	3.63E-2	1.17 (8586,1841,1220,307)	[+] Show genes
GO:0031982	vesicle	5.05E-4	4.04E-2	1.17 (8586,1792,1220,299)	[+] Show genes
GO:0005834	heterotrimeric G-protein complex	8.99E-4	6.84E-2	3.79 (8586,13,1220,7)	[+] Show genes
GO:1905360	GTPase complex	8.99E-4	6.51E-2	3.79 (8586,13,1220,7)	[+] Show genes

Species used: Mus musculus

The system has recognized 9823 genes out of 10280 gene terms entered by the user.
9823 genes were recognized by gene symbol and 0 genes by other gene IDs .
1226 duplicate genes were removed (keeping the highest ranking instance of each gene) leaving a total of 8597 genes.
Only 8586 of these genes are associated with a GO term.

The GOrilla database is periodically updated using the GO database and other sources.
The GOrilla database was last updated on Sep 3, 2016

This results page will be available on this site for one month from now (until Oct 3, 2016). You can bookmark this page and come back to it later.

'P-value' is the enrichment p-value computed according to the mHG or HG model. This p-value is not corrected for multiple testing of 1521 GO terms.

'FDR q-value' is the correction of the above p-value for multiple testing using the Benjamini and Hochberg (1995) method.
Namely, for the ith term (ranked according to p-value) the FDR q-value is (p-value * number of GO terms) / i.

Enrichment (N, B, n, b) is defined as follows:
N - is the total number of genes
B - is the total number of genes associated with a specific GO term
n - is the number of genes in the top of the user's input list or in the target set when appropriate
b - is the number of genes in the intersection
Enrichment = (b/n) / (B/N)

Genes: For each GO term you can see the list of associated genes that appear in the optimal top of the list.
Each gene name is specified by gene symbol followed by a short description of the gene

Back to the GOrilla main page
