# Supplementary material for: Genome maintenance and bioenergetics of the long-lived hypoxia-tolerant and cancer-resistant blind mole rat, Spalax: a cross-species analysis of brain transcriptome
Source: Sci Rep. 2016 Dec 9;6:38624. doi: 10.1038/srep38624 (PMC5146665; doi:10.1038/srep38624)
Supplement: Supplementary Dataset 1 [file srep38624-s2.doc]

**Genome maintenance and bioenergetics of the long-lived hypoxia-tolerant and cancer-resistant blind mole rat, *Spalax*: a cross-species analysis of brain transcriptome**

Assaf Malik,Vered Domankevich, Han Lijuan, Fang Xiaodong, Abraham Korol, Aaron Avivi, Imad Shams

**TableS1: Individuals, species, and RNA-Seq samples used in the study.**

| **issue** | **species** | **age** | **unit** | **group** | **sex** | **species/strain** | **lane-id** |
| --- | --- | --- | --- | --- | --- | --- | --- |
| **brain** | *Spalax* (set1) | 1 | years | adult | f | *galili* 2n=52 | 1b |
|  |  | 3 | years | adult | f | *galili* 2n=52 | 1b |
|  |  | 5 | years | adult | m | *galili* 2n=52 | 1b |
|  |  | 5 | years | adult | m | *galili* 2n=52 | 1b |
|  |  | 12 | years | old | f | *golani* 2n=54 | 1b |
|  |  | 17 | years | old | f | *galili* 2n=52 | 1b |
|  | Rat (set2) | 5 | months | adult | m | SD | 3b |
|  |  | 5 | months | adult | m | SD | 3b |
|  |  | 7 | months | adult | m | SD | 4b |
|  |  | 10 | months | adult | m | SD | 5b |
|  |  | 16 | months | old | m | SD | 6b |
|  |  | 24 | months | old | m | SD | 2b |

*Age* column: As *Spalax* cannot be bred in captivity, they are captured in the field, and only 1-year old animals or older can be discriminated, ages of all *Spalax* individuals were determined by the time of their capture until sacrificed. RNA-Seq machine lane ids: 1b (L8_120408), 2b (L2_120611), 3b (L3_120608), 4b (L8_120611), 5b (L8_120606), 6b (L1_120611).
